# Low-intensity extracorporeal shockwave therapy in patients with diabetic kidney disease: a matched cohort study

**DOI:** 10.1007/s11255-025-04379-4

**Published:** 2025-02-12

**Authors:** Sofus Valentin Vestersager, Sune Moeller Skov-Jeppesen, Knud Bonnet Yderstraede, Claus Bistrup, Boye L. Jensen, Lars Lund

**Affiliations:** 1 Department of Urology, Odense University Hospital, Sdr. Boulevard 29, 5000 Odense C, Denmark; 2 Department of Clinical Biochemistry, Herlev and Gentofte Hospital, Copenhagen University Hospital, Herlev, Denmark; 3 Steno Diabetes Center, Odense University Hospital, Odense, Denmark; 4 Clinical Institute, University of Southern Denmark, Odense, Denmark; 5 Department of Nephrology, Odense University Hospital, Odense, Denmark; 6 Department of Cardiovascular and Renal Research, Institute of Molecular Medicine, University of Southern Denmark, Odense, Denmark

**Keywords:** Low-intensity extracorporeal shockwave therapy, Diabetic kidney disease, Estimated glomerular filtration rate, Albumin creatinine ratio, Matched cohort study, Funen diabetes database

## Abstract

**Purpose:**

Low-intensity extracorporeal shockwave therapy (LI-ESWT) is a potential novel treatment against diabetic kidney disease (DKD). The present study investigates the longer term effects of LI-ESWT on kidney function in patients with DKD.

**Methods:**

This matched cohort study included 28 patients with DKD, who received six sessions of LI-ESWT. Patients were matched 1:5 with patients from the Funen Diabetes Database. Multivariable adjusted eGFR and ACR were analyzed using multilevel mixed-effects linear regression. The primary outcomes were ACR and eGFR measured at 3, 6, 12, and 18 month follow-up. Secondary analyses with patients stratified for sex, age, baseline eGFR, and baseline ACR were made for the multivariable adjusted values of eGFR and ACR.

**Results:**

No significant difference in multivariable adjusted ACR or eGFR was found at 18 months. The intervention group showed a non-significant decrease in adjusted eGFR (1.83 mL/min/1.73 m^2^ lower, *p* = 0.15) and ACR (14%, *p* = 0,56). Stratified results revealed lower eGFR in patients > 60 years 3.64 mL/min/1.73 m^2^, *p* = 0.03) and those with baseline ACR ≤ 300 mg/g (3.64 mL/min/1.73, *p* = 0.007).

**Conclusion:**

LI-ESWT did not demonstrate overall statistically significant effects on eGFR and ACR at 3, 6, 12, or 18 months. However, secondary analyses suggest possible effects in certain subgroups. Clinical studies with larger samples are needed to clarify the efficacy of LI-ESWT in specific DKD patient subgroups.

*Trial Registration* The trial was prospectively registered July 31, 2015, at ClinicalTrials.gov with registration number NCT02515461.

## Introduction

The incidence of diabetes mellitus (DM) is increasing worldwide with more than 592 million people expected to be diagnosed with DM in 2035 [1]. It is estimated that 33% of patients with type 1 DM and 50% of patients with type 2 DM will develop chronic kidney disease (CKD) during a lifespan [2]. Diabetic kidney disease (DKD) is defined as kidney disease in patients with DM in the absence of other renal diseases [3]. Diabetic kidney disease is the most common cause of end-stage renal disease (ESRD), and is associated with significantly increased morbidity and mortality [2, 4, 5].

Current treatment options for DKD include renin–angiotensin–aldosterone system (RAAS) blockade, sodium-glucose transport protein 2 (SGLT-2) inhibitors, and Glucagon-Like Peptide-1 (GLP-1) receptor agonists have been shown to reduce albuminuria and loss of glomerular filtration rate in humans in addition to decreased inflammation and fibrosis in the kidney [2, 6]. However, despite these improvements increased risk of ESRD persists, thus novel treatments for DKD are needed.

Low-Intensity Extracorporeal Shock-wave Therapy (LI-ESWT) is a non-invasive and safe procedure for renal application, with only mild acute side effects [7]. Shock-wave therapy is used for lithotripsy but also for regenerative purposes in the treatment of erectile dysfunction, wound healing, and tendinopathies [8–11]. Additionally, LI-ESWT may reduce myocardial fibrosis after acute myocardial infarction (AMI) in pigs and ameliorate myocardial inflammation after AMI in rats [12, 13].

The aim of the present study was to investigate the longer term effects of LI-ESWT on DKD. The objectives were to study the glomerular filtration barrier integrity by urinary-albumin creatinine ratio (ACR) and estimated glomerular filtration rate (eGFR) up to 18 months after LI-ESWT in patients with stage 3 CKD. The study was a follow-up on a previously published cohort of patients with diabetic kidney disease [7]. In the present approach, they were compared retrospectively with registered data from the Funen Diabetes Database (FDDB).

## Method

### Study design

**Fig. 1:**
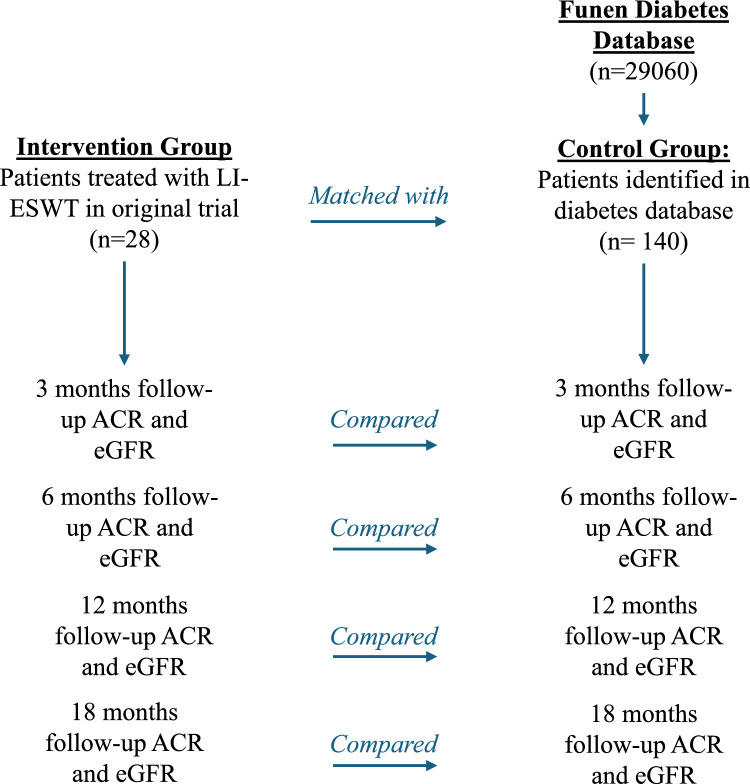
Study flow diagram

The study was designed as a matched cohort study with an intervention group and a matched control group. The intervention group consisted of patients with DKD who had been previously enrolled in a single-arm, interventional clinical study in which they received renal treatment with LI-ESWT directed to target the kidneys [14]. The matched control group consisted of patients with DKD who had been identified and followed in the FDDB. The intervention and control group match- and follow-up flow is illustrated in Fig. 1. Match parameters are shown in Table 1.

**Table 1:** Matching criteria used in FDBB

Sex ( =)
Age ($$\pm$$ 5 years)
eGFR ($$\pm$$ 10)
ACR ($$\pm$$ 10%)
Year of inclusion ($\pm$ 2 years)

eGFR was measured in mL/min/1.73 m^2^. ACR was measured in mg/g

### Intervention group

The recruitment and treatment protocol of the intervention group have previously been described [7, 14]. In short, 28 individuals aged 18–71 years were recruited for treatment with LI-ESWT between May 27th, 2015, and June 27th, 2019. All patients were recruited at a single center at Odense University Hospital, Odense, Denmark. The inclusion criteria for this study were a diagnosis of DM and concurrent eGFR between 30 and 60 mL/min/173 m^2^ (stage 3 CKD) measured on at least two occasions. Patients were excluded from the study if they met any of the following exclusion criteria: known or suspected non-diabetic kidney disease, kidney or urethral stone, obstructive uropathy, untreated urinary tract infection, kidney tumor, anticoagulant medical therapy, bleeding disorder, pregnancy, office blood pressure > 140/90 mmHg, abnormal renogram, single kidney, kidney transplant, acute myocardial infarction within 1 year, severe psychiatric disease, or pregnancy.

### LI-ESWT treatment sessions

The LI-ESWT treatment was conducted, as described previously using a Modulith SLX-2 device (Storz Medical, Tägerwilen, Switzerland) [7, 14]. Treatment was performed with patients placed in the supine position or laying on their side and each kidney was visualized by ultrasound. Water was used as a coupling agent between the Modulith SLX-2 and the skin. Each kidney received 1000 shocks at the upper pole, middle part, and lower pole, respectively. The first 200 shocks in each part of the kidney were given with gradually increasing energy levels, starting at 0.136 mJ/mm^2^ and increasing to 0.265 mJ/mm^2^. The remaining 800 shocks were given at 0.265 mJ/mm^2^. The shockwaves were administered with a frequency of 4 Hz and extended focal zone. Each patient received six treatment sessions over a period of 3 weeks, with a 3–4 day interval between each session.

### Endpoints

ACR and eGFR were measured at baseline and at 3 months, 6 months, 12 months, and 18 month follow-up in both groups.

### Control group and matching

Patients in the intervention group were matched in a ratio 1:5 with control patients identified in the FDDB. Matching criteria are described in Table 1, and include sex, age, ACR, eGFR, and year of inclusion. The FDDB is an ongoing database that includes patients with DM from the Funen area of Denmark. As of 2020, the database comprised 19.085 patients with type 2 DM and 5992 patients with type 1 DM. The FDDB was used to obtain values for ACR and eGFR for patients in the control group [15].

### Statistics

We used STATA version 18.0 (StataCorp, College Station, TX, USA) for statistical analyses. eGFR and ACR were compared between the intervention group and the control group at follow-up using multilevel mixed-effects linear regression. Treatment effect was adjusted for continuous covariates age, baseline ACR, and baseline eGFR. To investigate whether there was any variation in the treatment effect over time, we studied the interaction between treatment group and month of follow-up (3 months, 6 months, 12 months, and 18 months). The overall treatment effect was assessed across all visits in one model with eGFR as endpoint and another model with ACR as endpoint. To determine whether there was any difference in treatment effect over time, we investigated the interaction between treatment and month in separate models. Likelihood ratio test was applied to test for significant differences between a model without interaction between treatment and months versus a model including interaction between treatment and months. Random effects from match group and individual ID, allowing for random slope on month and unstructured covariance, were included in each model. For the fixed effects of each fitted model, we inspected the predicted values plotted against the observed values. Heteroscedasticity was assessed for the fixed effects by visual inspection of normal quantile plots and residuals plotted against predicted values. Best linear unbiased predictions (BLUPs) were predicted and inspected visually on a normal quantile plot for each level of random effect. We detected major deviations from normality assessing the distribution of residuals and BLUPs for the prediction of ACR. These deviations were attenuated by logarithmic transformation of ACR-values. The treatment effect size on ACR was determined by G-estimation with 100 bootstrap replications of the mean ratio between predicted ACR in non-treated patients and predicted ACR in treated patients with 95% confidence interval. G-estimation is a method where we use the parameters obtained from the mixed model to calculate the predicted outcomes if all patients had received treatment compared to if no patients had received treatment [16]. Subsequent bootstrap replication allows for estimation of a 95% confidence interval for the mean ratio. From the mixed model, predicted mean ACR in non-treated patients was calculated as the exponential value of the constant term, whereas predicted mean ACR in treated patients was calculated as the exponential value of the treatment effect parameter added to the constant term. No other deviations from model assumptions were found. Plotted predicted eGFR versus observed eGFR and predicted log-ACR versus observed log-ACR showed good model fit.

## Results

Baseline patient characteristics and demographics in the intervention group and the control group are described in Table 2. At baseline, there were no significant differences between the intervention group and the control group regarding sex, age, and year of inclusion.

**Table 2:** Baseline comparison of the intervention group and the control group

	Intervention group	Control group	*p* value
*Sex*			1.00
Male	20 (71.43%)	100 (71.43%)	
Female	8 (28.57%)	40 (28.57%)	
*Age*	61 (7.98)	62 (8.33)	0.48
*Year of inclusion*	2017 (1.38)	2017 (1.29)	0.82
*Baseline eGFR*	39.4 (11.1)	42.3 (11.9)	0.11
*Baseline ACR*	498 (739)	435 (674)	0.7

Data are number (%) or mean (sd). eGFR was measured in mL/min/1.73 m^2^. ACR was measured in mg/g. Two-sample *T* test assuming unequal variance was used to calculate the *p* value

### Baseline eGFR and ACR

We detected no significant differences between the intervention group and the control group at baseline (Table 2). Mean eGFR was numerically, but not statistically lower in the intervention group at baseline where eGFR was 39.4 mL/min/1.73 m^2^ (SD 11.1) in the intervention group compared to 42.3 mL/min/1.73 m^2^ (SD 11.9) in the control group (*t* test for difference between groups, *p* = 0.11). At baseline, mean ACR was 498 mg/g (SD 739) in the intervention group versus 435 mg/g (SD 674) in the control group (*t* test for difference between groups, *p* = 0.70).

### Unadjusted eGFR at follow-up

**Fig. 2:**
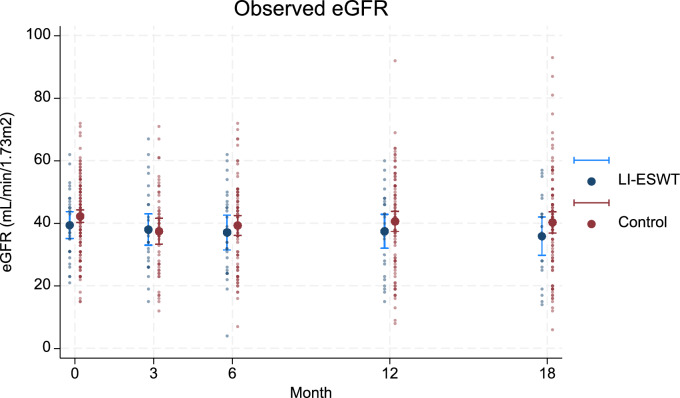
Individual unadjusted eGFR together with mean unadjusted eGFR with 95% CI in the intervention group versus the control group plotted at baseline, 3 month follow-up, 6 month follow-up, 12 month follow-up, and 18 month follow-up

In the intervention group compared to the control group, mean unadjusted eGFR was 38.0 mL/min/1.73 m^2^ (SD 12.9) versus 37.5 mL/min/1.73 m^2^ (SD 14.3) at 3 month follow-up, 37.0 mL/min/1.73 m^2^ (SD 14.0) versus 39.3 mL/min/1.73 m^2^ (SD 14.0) at 6 month follow-up, 37.4 (SD 13.1) versus 40.6 mL/min/1.73 m^2^ (SD 15.4) at 12 month follow-up, and 35.9 mL/min/1.73 m^2^ (SD 13.5) versus 40.3 mL/min/1.73 m^2^ (SD 16.7) at 18 month follow-up (Fig. 2).

### eGFR at follow-up adjusted for sex, age, baseline eGFR, and baseline ACR

Overall, multivariable adjusted eGFR was nonsignificantly lower (1.83 mL/min/1.73 m^2^ (95% CI: − 0.63; 4.28, *p* = 0.15) at follow-up in the intervention group compared to the control group. There was no significant interaction between treatment and month of follow-up (*p* = 0.63). Data are summarized in Table 3.

**Table 3:** Multivariable adjusted differences in eGFR and ACR at follow-up

	3 months	6 months	12 months	18 months	Overall
eGFR (mL(min/1.73 m^2^)	2.38 (− 0.53; 5.29)	1.64 (− 1.27; 4.56)	1.30 (− 2.32; 4.92)	3.32 (− 1.46; 8.11)	1.83 (− 0.63; 4.28)
ACR (%)	− 27 (− 29; 83)	− 8 (− 45; 63)	11 (− 69; 91)	− 33 (− 20; 86)	14 (− 28; 57)

Numbers show the mean difference with 95% CI between the intervention group compared to the control group

### Unadjusted ACR at follow-up

**Fig. 3:**
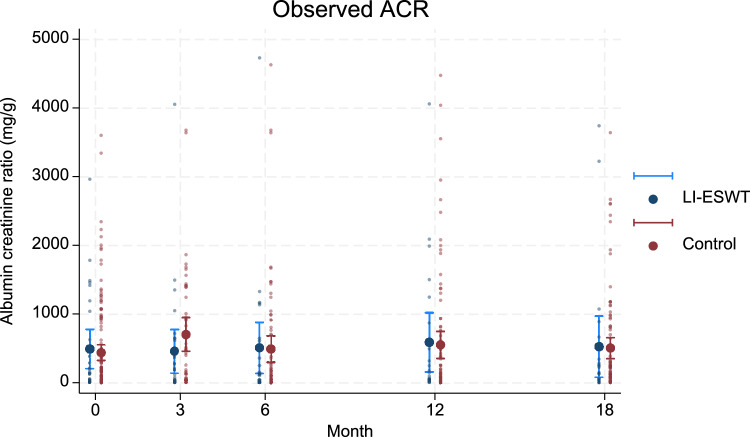
Individual unadjusted ACR together with mean unadjusted ACR with 95% CI in the intervention group versus the control group plotted at baseline, 3 month follow-up, 6 month follow-up, 12 month follow-up, and 18 month follow-up

In the intervention group compared to the control group, unadjusted mean ACR was 460 mg/g (SD 816) versus 702 mg/g (SD 855) at three months follow-up, 511 mg/g (SD 939) versus 491 mg/g (SD 845) at 6 month follow-up, 591 mg/g (SD 996) versus 552 mg/g (SD 935) at 12 month follow-up, and 527 mg/g (SD 1002) versus 505 mg/g (SD 737) at 18 month follow-up (Fig. 3).

### ACR at follow-up adjusted for sex, age, baseline eGFR, and baseline ACR

Overall, multivariable adjusted ACR was nonsignificantly lower (14% 95% CI: − 28; 57, *p* = 0.56) lower at follow-up in the intervention group compared to the control group. For ACR, there was no significant interaction between treatment and time (*p* = 0.90). Data are summarized in Table 3.

### Stratified analyses: eGFR

Notably, there was a significant difference in eGFR at follow-up in two patient subgroups: In patients aged > 60 years, multivariable adjusted eGFR was 3.2 mL/min/1.73 m^2^ (95% CI: 0.37; 6.02, *p* = 0.03) lower at follow-up in the intervention group compared to the control group and in patients with baseline ACR ≤ 300 mg/g, multivariable adjusted eGFR was 3.64 mL/min/1.73 m^2^ (95% CI 1.01; 6.2, *p* = 0.007) lower at follow-up in the intervention group compared to the control group. Data are summarized in Table 4.

**Table 4:** Stratified analysis for multivariable adjusted eGFR

	eGFR (ml/min/1.73 m^2^)	*p* value
*Sex*		
Female	− 2.08 (− 1.83; 5.99)	0.30
Male	− 1.58 (− 1.43; 4.58)	0.30
*Age*		
≤ 60 years	− 0.62 (− 3.43; 4.68)	0.76
> 60 years	− 3.2 (0.37; 6.02)	0.03
*eGFR at baseline*		
≤ 40 mL/min/1.73 m^2^	− 2.22 (− 0.86; 5.30)	0.16
> 40 mL/min/1.73 m^2^	− 1.60 (− 2.40; 5.60)	0.43
*ACR at baseline*		
≤ 300 mg/g	− 3.64 (1.01; 6.2)	0.007
> 300 mg/g	1.08 (− 3.20; 5.35)	0.62

Data are presented as the overall mean difference with 95% CI in multivariable adjusted eGFR in the intervention group compared to the control group

### Stratified analyses: ACR

Predicted ACR was lower across all stratified groups, except in patients with ACR ≤ 300 mg/g, where predicted ACR was 2% higher at follow-up in the intervention group compared to the control group. However, none of the results were statistically significant. Data are summarized in Table 5.

**Table 5:** Stratified analysis for multivariable adjusted ACR

	ACR (%)	*p* value
*Sex*		
Female	− 8 (− 50; 66)	0.77
Male	− 15 (36; 67)	0.94
*Age*		
≤ 60 years	− 12 (− 46; 69)	0.87
> 60 years	− 10 (− 56; 75)	0.92
*eGFR at baseline*		
≤ 40 mL/min/1.73 m^2^	− 18 (− 41; 77)	0.97
> 40 mL/min/1.73 m^2^	− 14 (− 41; 69)	0.63
*ACR at baseline*		
≤ 300 mg/g	2 (− 40; 44)	0.35
> 300 mg/g	− 2 (− 38; 43)	0.96

Data are presented as the overall mean difference with 95% CI, in percentage, between multivariable adjusted ACR in the intervention group compared to the control group

## Discussion

In the present study, we found no significant differences in ACR and eGFR between the intervention and control group. In stratified analyses, no differences were found except in patients > 60 years and those with baseline ACR ≤ 300 mg/g, where eGFR at follow-up was lower in the intervention group compared to the control group. All point estimates in the stratified analyses indicated lower ACR in patients treated with LI-ESWT, except in patients with low ACR at baseline, consequently slower progression toward ESKD can be anticipated in patients treated with LI-ESWT. Our data indicate that in a small cohort of well-characterized patients with DKD, LI-ESWT has no overt therapeutic effect on glomerular filtration rate or glomerular barrier integrity. However, LI-ESWT may have a beneficial effect on ACR, although larger studies may be needed to clarify its role in lowering ACR.

LI-ESWT has shown beneficial renal effects in both pig and rat models, by inducing various angiogenic factors and reducing inflammation [17, 18]. The shockwaves have a short duration of 10 μs and are characterized by a rapid positive pressure, followed by a period of negative pressure, ending with a return to ambient pressure, and work by forming cavity bubbles in the target tissue [19]. The subsequent stress induced by shockwaves leads to the activation of cell surface proteins, such as caveolin-1 and β1-integrin, which act as mechanotransducers [20]. These mechanotransducers convert physical stimuli into biochemical signals that may improve renal angiogenesis through upregulation of VEGF and eNOS [21]. In rats with CKD, LI-ESWT treatment increases the expression of VEGF and eNOS, thereby preserving residual renal function [22]. Furthermore, in rats with DKD, 6-week LI-ESWT treatment increases podocyte regeneration, renal cell proliferation and reduces diabetes-induced renal inflammation, and urinary-albumin excretion [18]. Damage to podocytes leads to an increase in albuminuria and a decline in renal function in patients with DKD [23]. LI-ESWT, by promoting podocyte regeneration, may prove to ameliorate albuminuria with less treatment related morbidity than drugs currently in use [18].

The current intervention for CKD aims to slow the progressive decline in kidney function [24]. We tested LI-ESWT as a novel treatment for DKD with the potential to improve renal function by increasing pro-angiogenic factors, enhance endothelial cell proliferation, vascularization, and perfusion in renal tissue while reducing renal inflammation and fibrosis, as demonstrated in animal studies [25]. However, it is unclear whether LI-ESWT have the same beneficial effects in human diabetic individuals.

Patients in the intervention group showed stable levels of hemoglobin A1c and arterial blood pressure at follow-up, indicating patient adherence to anti-diabetic and antihypertensive regimens, making it unlikely to have influenced the outcome of ACR and eGFR [14]. However, since hemoglobin A1c and blood pressure were not recorded in patients in the control group, we cannot exclude that some time-dependant variables such as hypertension may have acted as potential confounders.

DKD is diagnosed in patients with diabetes either by renal biopsy or clinically by an albumin/creatinine ratio (ACR) ≥ 30 mg/g and/or sustained reduction in estimated glomerular filtration rate (eGFR) ≤ 60 mL/min per 1.73 m^2^ [26]. Some patients with DKD have normal urinary-albumin levels and can be diagnosed with normoalbuminuric DKD; however, this study did not include any patients with normoalbuminuric DKD [27]. Renal biopsy is the gold standard for the diagnosis of DKD and should be considered if other kidney diseases are suspected [3]. All patients in the intervention group were diagnosed by either kidney biopsy or a sustained reduction in eGFR. In contrast, in the FDDB, patients were selected for inclusion in the control group based on a single measurement of eGFR < 60 mL/min/1.73 m^2^. Consequently, patients with a single fluctuation in eGFR may have been included in the control group. Thus, our comparison between the groups may have been biased toward a null-effect of LI-ESWT or a less favorable outcome in the intervention group. In the present study, eGFR was based on plasma creatinine measurements instead of the Tc-99 m-DPTA clearance method, which was available and previously published in the intervention group [7]. However, GFR determined with Tc-99 m-DPTA clearance method was not available for patients in the FDDB. The use of eGFR based on plasma creatinine instead of GFR, may have resulted in less accurate estimates of the kidney function. Likewise, ACR was calculated based on a spot urine sample instead of a 24 h urine collection.

We observed considerable variation between the patients included in the present study, especially in terms of ACR. In particular, this study indicates that LI-ESWT may work better in patients with higher ACR. Although this study found no overall significant differences in multivariable adjusted ACR and eGFR between the treatment and control groups, multivariable adjusted eGFR was found to be lower in patients in the intervention group aged > 60 years and in patients with ACR ≤ 300 mg/g compared to the control group. This may indicate that LI-ESWT has no therapeutic effect but may be potentially harmful in patients with a milder degree of kidney disease and in older patients. On the other hand, a reduction in eGFR is an anticipated consequence of initiating an antihypertensive therapy; however, this decline is not associated with an increased long-term risk of renal function decline in patients with DKD [28]. Our findings could also be due to residual confounding of lower eGFR in the intervention group compared to the control group at baseline. Thus, further studies are needed to assess the efficacy of LI-ESWT in different subgroups of patients with DKD.

### Strengths and limitations

To account for changes in the standard treatment regimen of DKD, one of the matched variables used was date of inclusion. Importantly, Finerenone as a new treatment for DKD, was first approved in the European Union after the end of this study [29].

Additional variables, such as diabetes mellitus type, medication use, and blood pressure, could have been drawn from Danish databases and incorporated into the adjusted analysis. However, due to the limited size of the study population, it would not have been statistically meaningful to adjust for further variables in the stratified analysis.

There was considerable heterogeneity in albumin excretion between the patients in both the intervention group and the control group at baseline. It is possible that LI-ESWT has variable effects on selected subgroups of patients, which may be further clarified in other studies. The present study included 28 participants in the intervention group, which did not allow for a primary analysis of specific subgroups. Controlling for anti-diabetic medication use, including GLP-1 agonists and SGLT-2 inhibitors, may be of interest in further studies. Further studies are needed to examine dose–response effects of LI-ESWT and determine the optimal intensity and interval between LI-ESWT treatment regimes.

## Conclusion

This study found none overall statistically significant beneficial effect of LI-ESWT treatment on renal function in patients with DKD, determined by ACR and eGFR. Specific subgroups of patients with DKD may have less favorable outcomes in eGFR after LI-ESWT. In most subgroups of patients, ACR tended to be lower after LI-ESWT, and further clinical studies are needed to evaluate the role of LI-ESWT in the treatment of DKD.

## Data Availability

The datasets analyzed during the current study are not publicly available due to the privacy of study participants. Researchers interested in data from the Funen Diabetes Database can contact Steno Diabetes Center Odense, email: ouh.sdco@rsyd.dk.
